# Comparative Analysis of *Ascaris suum* and *Macracanthorhynchus hirudinaceus* Infections in Free-Ranging and Captive Wild Boars (*Sus scrofa*) in Hungary

**DOI:** 10.3390/ani14060932

**Published:** 2024-03-18

**Authors:** Csaba Farkas, Alexandra Juhász, Balázs Fekete, Borisz Egri

**Affiliations:** 1 Department of Animal Science, Albert Kázmér Faculty of Mosonmagyaróvár, Wittmann Antal Multidisciplinary Doctoral School, Széchenyi István University, H-9200 Mosonmagyaróvár, Hungary; 2 Tropical Disease Biology, Liverpool School of Tropical Medicine, Liverpool L3 5QA, UK; 3 Institute of Medical Microbiology, Semmelweis University, H-1089 Budapest, Hungary; 4 Nexum Veterinary Medicine and Service Ltd., H-2440 Százhalombatta, Hungary

**Keywords:** *Ascaris suum*, endoparasites, *Macracanthorhynchus hirudinaceus*, zoonosis

## Abstract

**Simple Summary:**

In total, 216 wild boars (*Sus scrofa*), a known host of roundworm (*Ascaris suum*) and giant thorny-headed worm (*Macracanthorhynchus hirudinaceus*), were examined. The infection rates of the free-ranged and captive populations were compared from June 2015 to June 2023 in Hungary. Of the 173 dissected wild boars from the wild, 57 (32.9%) were infected with *A. suum*, while 30 (69.8%) of the 43 individuals from the captive area were infected. The prevalence of *M. hirudinaceus* in the wild population was 9.25% (16 wild boars), while that of the captive animals was 34.89% (15 wild boars). The prevalence of *A. suum* in the entire population was 40.28% (87 wild boars), while the prevalence of *M. hirudinaceus* was 14.35% (31 wild boars). In the case of the examined helminths, the captive herd was 36.9% more infected than the herd living in the open area.

**Abstract:**

*Ascaris suum* and *Macracanthorhynchus hirudinaceus* cause a large loss of yield in farm animals as well as in free-living and captive wild boar herds, thereby causing economic damage. This study compared *A. suum* and *M. hirudinaceus* infections in free-ranging and captive wild boars (*Sus scrofa*) in Hungary. The authors measured the *A. suum* and *M. hirudinaceus* infections of a 248-hectare wild boar garden and an 11,893-hectare free-living wild boar herd in the sample area. In all cases, samples were collected from shot wild boars. In total, 216 wild boars were examined from June 2015 to June 2023 in Hungary. Of the 173 dissected wild boars from the wild, 57 (32.9%) were infected with *A. suum*, while 30 (69.8%) of the 43 individuals from the captive area were infected. The prevalence of *M. hirudinaceus* in the free-living area population was 9.25% (16 wild boars), while that of the captive population was 34.89% (15 wild boars). In the case of the examined helminths, the captive herd was 36.9% more infected than the herd living in the open area.

## 1. Introduction

Although the helminth parasites of domestic pigs are well documented, little information is available about the intestinal helminth infections of wild boar *Sus scrofa* (Linnaeus, 1758) [1,2,3].

Similar to in other European countries, in Hungary, wild boar are the most widespread game species [4]. An increasing wild boar population will lead to a rise in the potential for zoonotic disease transmission, therefore health surveys of these animals are of importance [5]. Despite this, information on the intestinal helminth fauna of the wild boar is still scanty and fragmentary in Hungary. Takacs’s paper was the first and still the most comprehensive publication dealing with the helminth fauna of wild boars in Hungary [6]. According to Egri and Sugár, *A. suum* larvae and adults are usually found in wild boars that are older than six months [7]. Regarding *M. hirudinaceus* and *A. suum*, the only study on wild boars in Hungary was published by Farkas et al. in 2021 [8] in which their levels in captive and free-living wild boars were determined in the cases of 76 individuals.

Changes in human habitation to suburban areas, the increased use of lands for agricultural purposes, increased hunting activities and the consumption of wild boar meat have increased the chances of exposure of wild boars to domestic animals and humans [5]. In addition, the recreational hunting of wild boar and the consumption of wild boar meat has provided ample opportunity for direct human contact with wild boar in some regions of the world, creating an ideal environment for the transmission of pathogens between wild boar and domestic pigs and between wild boar and humans [9]. In some regions, wild boar populations are increasing, partly due to the development of a commercial hunting industry [10], which could further exacerbate the problem.

Wild boars are known reservoirs for *Ascaris suum* (Goeze, 1782) (Ascaridida, Ascarididae), which is transmissible to domestic pigs and humans. This is a widely spread intestinal parasite, and a persistent problem. The infection may have a mild to moderate adverse effect on the wild boar’s health and weight gain. Adult worms have mechanical and toxic effects that selectively extract certain nutrients in wild boars [11,12]. The development and survival of worms in the environment depend on many abiotic and biotic factors. The eggs of *A. suum*, deposited in soil, can survive for up to 10 years. The eggs are very resilient and can survive extreme environmental conditions such as frost and extreme heat. Therefore, it is virtually impossible to completely remove *A. suum* eggs from the environment where an infected animal has been present. The type of farming determines the rate of transmission and the risk of economic losses due to parasitism [13]. For example, in organic pig farms, all ages are constantly exposed to *A. suum*, but younger animals are mainly infected. Conducting long-term pasture rotation to eliminate contamination is not possible, and control programs should therefore include thorough cleaning in the barn and composting the long-term-stored bedding material and manure to inactivate the eggs and reduce transmission to pigs [14]. *Ascaris suum* is important in wild boars in game enclosures that are kept constantly in close proximity to one site and come into contact with contaminated bedding, and are thus most severely infected [15]. In game parks, there are more cases of higher parasitic infection, because the animals are kept in one place, and the egg density is higher in soil than in free-living environments [16].

The other common helminth species in wild boars is *Macracanthorhynchus hirudinaceus* (Pallas, 1781) (Acanthocephala, Oligacanthorhynchidae). In the case of the *M. hirudinaceus*, human infection has also been reported several times [17]. This parasite penetrates the intestinal mucosa with its proboscis-shaped head part armed with hooks and then causes perforations and/or ulcerations of the intestinal wall. The definitive hosts become infected by eating intermediate hosts, which are always an insect with infective cystacanth larvae. The most effective way to protect against intermediate hosts is to disinfect the soil in wild boar gardens and organic pig farms. In terms of infection by age group, the risk of infection is higher for individuals older than one and a half years [7]. Eggs resulting from the sexual reproduction of adult individuals that developed in the host are released into the outside world with the host’s feces. Nutrients are absorbed (similarly to tapeworms) through their transversely ringed tegument [18,19]. The conquest of the giant thorny-headed worm and its spread throughout the world continues to this day. High prevalences of *M. hirudinaceus* in wild boars have been recorded.

The zoonotic aspects of *A. suum* and *M. hirudinaceus* are important; several sporadic human cases have been reported. There are also reports of human infections of *M. hirudinaceus* from outside the Asian region; for example, the infection was detected in Peru in a 45-year-old man [17,20,21,22,23].

Despite the large population and wide distribution of wild boars in Hungary, relatively little is known about their endoparasites. We examined 216 carcasses of wild boars, to have a better understanding of their population distribution in a captive versus wild population in Hungary.

## 2. Materials and Methods

### 2.1. The Sample Area

**Figure 1:**
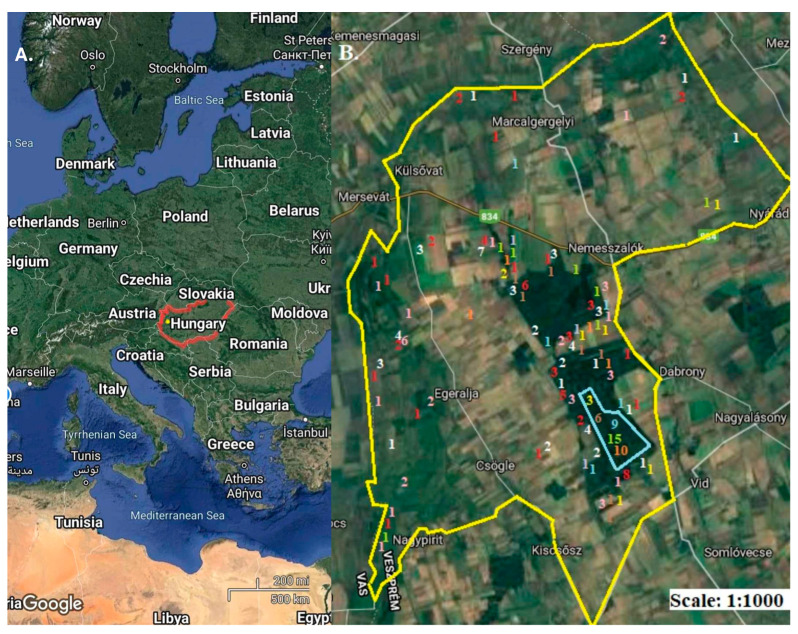
(**A**) Location of Hungary in Central Europe. The yellow dot marks the location of the hunting area. (**B**) The 11,893-hectare hunting area with the marking of the wild boar garden, which is 248 hectares. On the map, the numbers represent the number of wild boars shot at the marked location; yellow for 2015, brown for 2016, blue for 2017, green for 2018, orange for 2019, purple for 2020, and white for 2021, with red numbers indicating the drop locations of individuals shot in 2022 and pink for 2023. (Source: maps.google.com, accessed on 14 February 2024).

To find *A. suum* and *M. hirudinaceus* parasites, 216 wild boars were investigated via necropsy between June 2015 and June 2023 in the area of the Marcal-Bitvaközi Hunting Company (Figure 1). We originally planned to collect samples from the same number of wild boars from captive and free-ranged areas between 2015 and 2023. Unfortunately, in order to prevent the spread of African Swine Fever, the authorities suspended the operation of wild boar gardens and wild boar farms. The area is located at the coordinates 47°15′34″ N; 17°11′53″ E and 47°17′48″ N; 17°22′49″ E. There are smaller villages in the hunting area, and the people living there often work in and travel through the forest and agricultural areas. In this hunting area, only the Marcal-Bitvaközi Hunting Company can carry out game management activities; there is no game farm operated by anyone else in the area. The wild boar garden under examination is located in the south-eastern part of the area, in the part of two large forest blocks in the area close to Dabrony. Within the forest blocks is intensive wild land management; the water supply is ensured with the help of solar wells, and there is hardly any hunting activity, so that the game can find food, water and peace. Hunting activity is concentrated on the edges of the forests and the borders of agricultural areas, in order to reduce the damage caused by wild animals to crops. The concentrated hunting activity facilitated the sample collection. This wildlife management area is dominated by big game and, temporarily, big game areas. At the end of each hunting year, the professional staff of the Hunting Company estimates the number of wild animals living in the area and prescribes a shooting plan based on this. In all cases, the samples were collected in accordance with the regulations of the Hunting Authority, as well as the annual preliminary stock estimate and shooting plan, from game shot during the hunting season, during the regular operation of the Hunting Company. Exclusively wild boars killed in the manner mentioned here were sampled. Accordingly we examined the carcasses of 216 wild boars in order to better understand their stock distribution in the captive and wild populations in Hungary.

The moist soil, wallows and puddles attract wild boar to the area. In periods of drought, a continuous water supply must be ensured with the help of solar wells.

Bitva and Marcal, in addition to being the rivers of greatest importance in crossing the territory, the Hajagos, Körös and Szalóki streams and the Hunyor stream, which functions as a channel, flow through the area. There are also boggy, marshy areas in the floodplains of larger watercourses, covering thousands of hectares. During periods of rainfall and floods, the areas flooded with water made it difficult to collect samples, as it is almost impossible to drive in these parts of the area. The technologies of the wildlife management for the captivity and natural areas are almost identical. Both technologies involve continuous feeding, wildlife management and water supply.

### 2.2. Sample Collection

The animals were apparently healthy, wild boars that were shot during regular hunting and were provided for the survey by hunters. The visceras were removed on the hunting grounds, and the small intestines of the shot animals were investigated in the field. During the documentation, we recorded the epidemiological data of the animal. The helminthological determination of the worm species was carried out in the laboratory of the Animal Health Unit of the Animal Science Department of the Albert Kázmér Mosonmagyaróvár Faculty of Széchenyi István University and in the properly equipped veterinary clinic of Nexum Veterinary Medicine and Service Ltd. in Százhalombatta (Hungary).

In all cases, sampling began with the removal and separation of the viscera. The stomach and small intestine were excavated and washed separately along their entire length [19]. The entire contents of the intestine and the stomach were filtrated through a sieve with a hole size of 1 mm × 1 mm. The parasites were collected by hand from the solution; they were then stored in glass containers containing a pre-prepared solution containing 90% ethanol and 5% glycerol and labeled with an identification number. All samples were collected during legal hunts; therefore, no ethical approval was requested.

**Figure 2:**
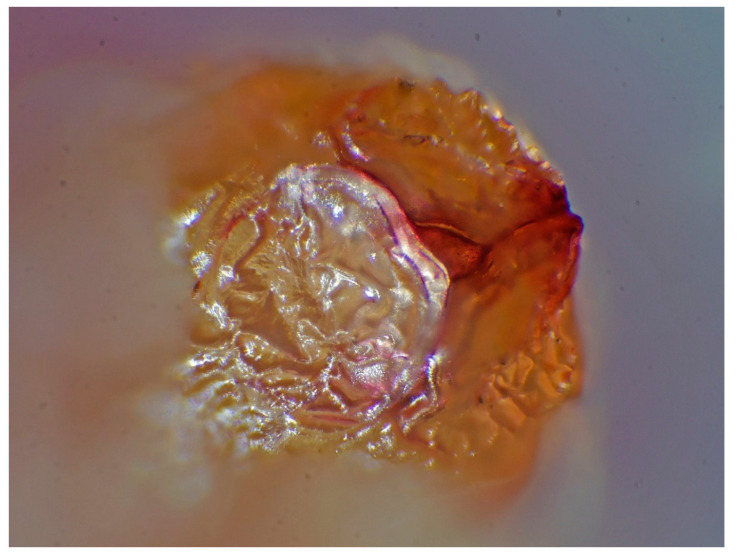
Mouth orifice of *Ascaris suum* with three swollen lips (×8) (original).

**Figure 3:**
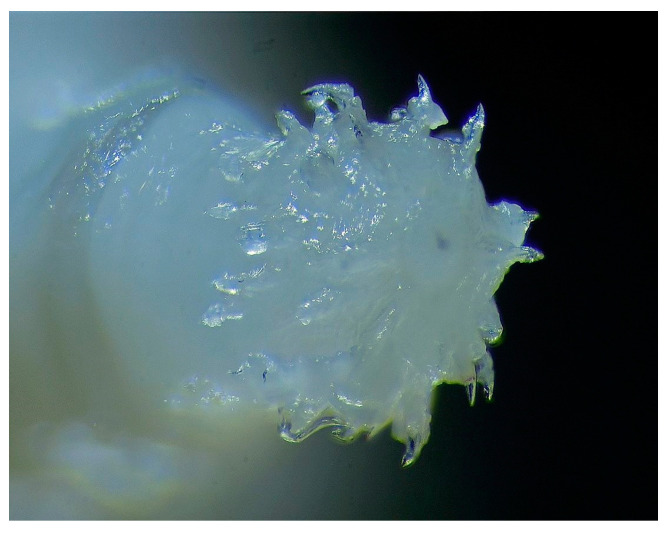
Mouth orifice of *M. hirudinaceus* with tiny hooks (×8) (original).

**Figure 4:**
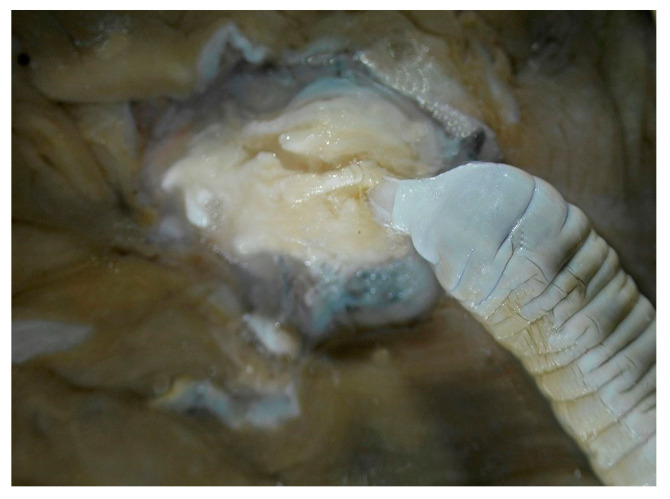
Penetration of *M. hirudinaceus* into the intestinal mucosa (×4) (original).

Samples were stored in a refrigerator at 4 °C until they were processed. Worms were identified according to the morphological features described by Kassai in 2003 [18]. The samples were identified using a PZ0 MST131-type and a Zeiss Ergaval and a Zeiss Discovery V8 stereo microscopes (Carl Zeiss Technique Ltd., Budapest, Hungary). The photos were taken with a Panasonic DMC-G6 camera connected to a Zeiss Discovery V8 stereomicroscope, with eight-times magnification, which realized three-dimensional pictures of the examined parasites (Figure 2, Figure 3 and Figure 4).

### 2.3. Statistical Methods

The SPSS 29 software was used for statistical calculations. The *X*^2^ test was used to calculate the relative infection rate of wild boars in free-ranging and captive areas. The numerical value of this ratio is given by Cramer’s V index. The average number of parasites per infected animal was calculated using the Shapiro–Wilk test and a Q–Q graph. Based on the results of these tests, we used the Mann–Whitney U test and Mood’s median test to calculate the intensity of the infection difference between the free-ranging and captive herds. The Mann–Whitney U test and the Kruskal–Wallis test helped us to calculate whether the animal’s maintenance technology conditions influence the degree level of infection, the average number of parasites per infected animal or the number of parasites per animal infected with both parasites.

## 3. Results

In order to calculate the effect of the high stocking density in the captive area or the lower stocking density in the free-ranged area on the level of infection, we used the chi-square test during our investigations. The results of the current investigation, as well as those of previous ones [8], show that infection in the captive area and non-infection in the free-living areas are dominant. The main quantitative parasitological results for wild boar stocks held in free and captive areas are contained in Table 1. The proportion of infection depends on the animal being dropped in a closed or freely managed area. The value of the *X*^2^ test was x^2^(1) = 19.409, the empirical significance was at *p* < 0.001, and the value of Cramer’s V indicator, which indicates the strength of the relationship was 0.300, *p* < 0.001. In the captive area, the prevalence of examined and shot game under investigation was 69.8%, which is 36.9% higher than the prevalence of wild game living and shot in free areas, which is only 32.9%. In calculating the average number of parasites per infected animal, it is clear that this indicator is higher for captive animals (5.5 helminths/individual) than for free-living herds (4.11 helminths/individual). The number of infections in our examinations (Shapiro–Wilk test and Q–Q graph) does not follow a normal distribution. Based on this result, we used the Mann–Whitney U test and Mood’s median test. Based on the results of both tests, it can be stated that the maintenance technology of the herds has a great influence on the intensity of the infection. The intensity value was higher for the tested individuals of the herd of the captive area.

In considering the herds living in free-living and captive areas, the degrees of infection show a significant difference in the cases of the *A. suum* and *M. hirudinaceus* infections. For both parasite infections, the average number of parasites per animal was higher in wild boars living in captive areas than in wild boars living in free areas.

The average value of the number of infections per infected animal was examined. When calculating this, in the case of the *M. hirudinaceus* infection, we obtained a result that shows a significant difference between the herds living in the captive area and those in the free-living area.

The number of parasites per animal infected with both parasites was also subjected to statistical analysis. In the comparison of free and captive areas, the difference is significant with regard to the *A. suum* and *M. hirudinaceus* infections. The statistical results of our calculations are presented in Table 2.

The number of infected animals was also examined. The corresponding calculations were carried out using the Mood’s median test (Table 3). Wild boars killed and examined in the free-living area were less infected than those shot in the captive area. The difference is significant for the total number of infections: x^2^(1) = 5.395, *p* = 0.020. In the case of *A. suum* infection, this difference is not significant (x^2^(1) = 0.635, *p* = 0.425), while it is significant in the case of *M. hirudinaceus* infection (x^2^(1) = 4.121, *p* = 0.042).

## 4. Discussion

Based on our studies, it can be concluded that the high population density in the captive wild boar population and the fact that parasitic eggs from the feces remain in the soil for a long time are the principal reasons of re-infection being much higher than in the fee-living wild boars that frequently visit new areas. Wild boars kept in captive areas are gathered in a relatively small area, which manifests the increased risk of outbreaks of diseases, including zoonosis. In captivity, they almost all visit the same feeding and wallowing places. Moreover, their places of rest in the daytime are also the same. This makes direct or indirect contact with each other much more active than in free-living wild boars.

During our examinations, it was clearly established that in the case of the two parasite species examined (*A. suum*, *M. hirudinaceus*), the degree of infection was much higher due to the high stocking density of the captive stock due to the husbandry conditions. However, it should not be disregarded in the case of stocks managed in the free-living area.

Both examined parasite species are widespread in many parts of the world and continue to spread to this day. Based on our results, it can be said that the *A. suum* infection in Hungary (40.28%) is much higher than, for example, in Russia (37.28% ↓), Korea (36.48% ↓), Iran (35.52% ↓), Nepal (33.28% ↓), Brazil (32.99% ↓), parts of Serbia around Belgrade (30.91% ↓), Rwanda (29.68% ↓), Cameroon (28.68% ↓) and Sicily (23.68% ↓). The difference is smaller, but also negative, in Serbia (11.25% ↓), some areas of Moldova (21.88% and 17.68% ↓), Argentina (17.28% ↓) and Mexico (8.28% ↓). Compared to our test results, the higher prevalence of *A. suum* appeared only in Uganda (13.14% ↑) and Moldova, in the second examination area (4.32% ↑).

Although *M. hirudinaceus* infection occurs with a lower prevalence (14.35%) in Hungary compared to *A. suum*, it still appeared with a higher prevalence than in the results of most foreign tests. *M. hirudinaceus* was detected in Tunisia (47.35% ↑) and in two different areas of Iran with a particularly high occurrence (42.79% and 37.65% ↑). Studies from Argentina (18.65% ↑), Eastern Spain (6.35% ↑) and Turkey (4.65% ↑) also reported a slightly higher prevalence. *M. hirudinaceus* with the lowest occurrence was reported from Cameroon (14.15% ↓). It also occurred in small numbers in Brazil (13.31% ↓), Romania (12.69% ↓) and Moldova (12.95%, 11.55% and 1.95% lower infection prevalences), as well as in two areas of Serbia (4.9% and 6.82% ↓) and in Sicily (3.25% ↓). Taking into account our own results, there is hardly any difference with the test results in the Belgrade province of Serbia (1.85% ↓). The Sardinian studies showed the smallest differences in incidence; the difference was only −0.75% (Table 4, Table 5 and Table 6).

## 5. Conclusions

In consideration of all evidence presented here, we conclude that the *A. suum* and *M. hirudinaceus* infections were higher in the captive environment. The two nematodes are transmitted through the feco-oral route through contaminated feed, water or soil or an infected intermediate host and have the potential to accumulate in the soil of a captive environment. During their daily work and activities, people often eat in the area, where wild boars also walk and live, which increases the risk of zoonosis. Since *A. suum* has a direct life cycle and the ability to survive in the environment, there is a high possibility of environmental contamination being the reason for their higher prevalence in the captive herd. The regular feeding, hunting management and high population density in a small area are all stressful conditions for the animals. The constant stress of captivity makes animals more susceptible to parasitic infection as their immune system becomes weak. As no mortality or clinical signs were reported and the animals were apparently healthy during the study period, the high prevalence indicates a subclinical infection. Although the overall management of the captive animals, including nutrition and hygiene, was followed, this study shows that the Hunting Company’s management can be improved by re-standardizing or re-planning anthelmintic programs, as well as through quarantine treatment to prevent infection.

To decrease the prevalence of infection, it is recommended that the colony be prescribed with anthelmintics to treat *M. hirudinaceus* and *A. suum* infections. However, the medicinal treatment of wild populations is not risk-free in terms of ecotoxicology and resistance [46,47,48]. The treatment of wild animals with anthelmintics in their natural habitats requires further research due to the effects of anthelmintics on entomofauna. Residues of various parasiticides in the feces of treated animals have a non-targeted effect on the insects carrying the feces and on the decomposition processes of the feces [49,50]. However, it is acknowledged that the administration of drugs is challenging due to the population size and the boars’ ability to detect medication in their food [51]. Therefore, to reduce parasite infections, the long-term monitoring of the helminthiasis is required. This can be achieved through annual fecal examinations involving basic stool microscopy. The results will help to formulate a suitable deworming protocol for parasite control in these captive animals. Not all researchers agree with this method [52], but in our opinion, in the case of wild boar, the examination of feces is the most obvious approach, because the examination of the intestines of wild animals shot while hunting is not relevant in most cases. The examination of the intestines of shot game is usually conducted only for research purposes and to obtain an idea of the parasite infection of the individuals. However, feces are available all year round and can be collected, and the examination can be repeated several times to obtain more relevant information of the presence of parasites.

Wild boars try to live as naturally as possible but due to fecal contamination in public areas and their farming for meat, contact with humans is inevitable. Therefore, treatment with anthelmintics can be justified so as to avoid the zoonotic transmission of these parasites every way possible.

## Figures and Tables

**Table 1:** Quantitative parasitological results.

	*Ascaris suum*			*Macracanthorhynchus hirudinaceus*		
	Detected in Free-Living Area	Detected in Captive Area	Total	Detected in Free-Living Area	Detected in Captive Area	Total
Total number of examined animals	173	43	216	173	43	216
Number of infected individuals	57	30	87	16	15	31
Prevalence%	32.9	69.8	40.3	9.2	34.9	14.4
CI of prevalence (95%)	25.9–39.9	56.1–83.5	33.8–46.8	4.9–13.5	20.7–49.1	9.7–19.1
Mean intensity	3.56	2.80	3.30	1.94	5.40	3.61
CI of mean intensity (95%)	2.97–4.15	2.32–3.28	2.88–3.72	1.41–2.47	4.24–6.56	2.74–4.48
Median intensity	3.0	3.0	3.0	2.0	5.0	3.0
CI of median intensity (95%)	2.6–3.4	2.7–3.3	2.7–3.3	1.7–2.3	4.3–5.7	2.5–3.5
Number of all detected parasites	203	84	287	31	81	112
Minimum number of parasites in one infected animal	1	1	1	1	2	1
Maximum number of parasites in one infected animal	11	6	11	4	9	9

Quantitative parasitological results for *A. suum* and *M. hirudinaceus* in wild boars examined in the territory of the Marcal-Bitvaközi Hunting Company. The main quantitative parasitological characteristics of *A. suum* and *M. hirudinaceus* infections in free-living and captive wild boar herds.

**Table 2:** The results of our statistical calculations.

		Results of the Mann–Whitney U Test	Results of the Kruskal–Wallis Test
Calculation of the average number of infections per animal	*A. suum* infection	U = 2491.000	x^2^(1) = 14.299
		*p* < 0.001	*p* < 0.001
	*M. hirudinaceus* infection	U = 2660.500	x^2^(1) = 22.432
		*p* < 0.001	*p* < 0.001
	Total number of infections	U = 2165.000	x^2^(1) = 22.858
		*p* < 0.001	*p* < 0.001
Calculation of the average value of the number of infections per infected animal	*A. suum* infection	U = 714.000	x^2^(1) = 1.646
		*p* = 0.200	*p* = 0.200
	*M. hirudinaceus* infection	U= 562.000	x^2^(1) = 9.350
		*p* = 0.002	*p* = 0.002
	Total number of infections	U = 670.000	x^2^(1) = 2.777
		*p* = 0.096	*p* = 0.096
Calculation of the average number of infections per animal infected with both parasite species	*A. suum* infection	U = 43.500	x^2^(1) = 9.502
		*p* = 0.002	*p* = 0.002
	*M. hirudinaceus* infection	U = 14.500	x^2^(1) = 17.841
		*p* = 0.001	*p* < 0.001
	Total number of infections	U = 89.500	x^2^(1) = 1.488
		*p* = 0.232	*p* = 0.222

The results of Mann–Whitney U test and Kruskal–Wallis test.

**Table 3:** Results of Mood’s median test.

		Detected in Captive Area	Detected in Free-Living Area
*A. suum*	The median number of values above the median	10	24
	The number of values below the median	20	33
*M. hirudinaceus*	The median number of values above the median	15	16
	The number of values below the median	15	41
Total number of infections	The median number of values above the median	16	16
	The number of values below the median	14	41

Results of Mood’s median test (for quantitative parasitological characteristics) of shot on hunting in free-living and shot on hunting in captive wild boar populations.

**Table 4:** Research data on *A. suum* from different parts of the world.

Area	Maintenance Technology	Prevalence of *A. suum*	Year of Publication	Reference
Northern Iran	woodland area wild boar	4.76%	2018	[24]
Argentina	woodland area wild boar	23%	2019	[25]
Rwanda	pig farm	10.6%	2020	[26]
Denmark	captive wild boars	10.6%	2020	[27]
Moldova (Codrii)	woodland area wild boar	18.4%	2020	[28]
Moldova (Pădurea Domnească)	woodland area wild boar	44.6%	2021	[29]
Mexico	captive wild boars	32.2%	2021	[30]
Italy (Sicily)	woodland area wild boar	16.6%	2021	[31]
Serbia (Vojvodina)	captive wild boars	29.03%	2021	[32]
Serbia (Vojvodina)	woodland area wild boar	29.03%	2021	[32]
Serbia (Belgrade)	woodland area wild boar	9.37%	2022	[33]
Moldova	woodland area wild boar	22.6%	2022	[34]
Cameroon	pig farm	11.6%	2022	[35]
Uganda	pig farm	53.42%	2022	[36]
Korea	pig farm	3.8%	2022	[37]
Russia	woodland area wild boar	3%	2022	[3]
Brazil	woodland area wild boar	7.29%	2023	[38]
Nepal	woodland area wild boar	7%	2023	[39]

Research data on *A. suum* from different parts of the world.

**Table 5:** Research data on *M. hirudinaceus* from different parts of the world.

Area	Maintenance Technology	Prevalence of *M. hirudinaceus*	Year of Publication	Reference
Turkey (Bursa)	woodland area wild boar	19%	2011	[40]
Southwestern Iran	woodland area wild boar	52%	2016	[41]
Northern Iran	woodland area wild boar	57.14%	2018	[24]
Romania	woodland area wild boar	1.66%	2019	[42]
Northwestern Tunisia	woodland area wild boar	61.7%	2019	[43]
Argentina	woodland area wild boar	33%	2019	[25]
Moldova (Codrii)	woodland area wild boar	1.4%	2020	[28]
Moldova (Pădurea Domnească)	woodland area wild boar	2.8%	2021	[29]
Eastern Spain	woodland area wild boar	20.7%	2021	[44]
Italy (Sicily)	woodland area wild boar	11.1%	2021	[31]
Serbia (Vojvodina)	captive wild boars	9.45%	2021	[32]
Serbia (Vojvodina)	woodland area wild boar	7.53%	2021	[32]
Serbia (Belgrade)	woodland area wild boar	12.5%	2022	[33]
Moldova	woodland area wild boar	12.4%	2022	[34]
Cameroon	pig farm	0.2%	2022	[35]
Italy (Sardinia)	woodland area wild boar	13.6%	2022	[45]
Brazil	woodland area wild boar	1.04%	2023	[38]

Research data on *M. hirudinaceus* from different parts of the world.

**Table 6:** The relationship between the prevalences of *A. suum* and *M. hirudinaceus* from different parts of the world compared with our own research results.

	*A. suum*	*M. hirudinaceus*	Difference *A. suum*	Difference *M. hirudinaceus*	Reference
Our results (Hungary)	40.28%	14.35%			
Turkey (Bursa)	-	19%	-	+4.65%	[40]
Southwestern Iran	-	52%	-	+37.65%	[41]
Northern Iran	4.76%	57.14%	−35.52%	+42.79%	[24]
Rwanda	10.6%	-	−29.68%	-	[26]
Moldova area 1	18.4%	1.4%	−21.88%	−12.95%	[28]
Moldova area 2	44.6%	2.8%	+4.32%	−11.55%	[29]
Moldova area 3	22.6%	12.4%	−17.68%	−1.95%	[34]
Italy (Sicily)	16.6%	11.1%	−23.68%	−3.25%	[31]
Mexico	32%	-	−8.28%	-	[30]
Serbia area 1	29.03%	9.45%	−11.25%	−4.9%	[32]
Serbia area 2	29.03%	7.53%	−11.25%	−6.82%	[32]
Cameroon	11.6%	0.2%	−28.68%	−14.15%	[35]
Uganda	53.42%	-	+13.14%	-	[36]
Italy (Sardinia)	-	13.6%	-	−0.75%	[45]
Brazil	7.29%	1.04%	−32.99%	−13.31%	[38]
Nepal	7%	-	−33.28%	-	[39]
Argentina	23%	33%	−17.28%	+18.65%	[25]
Denmark	10.6%	-	−29.68%	-	[27]
Serbia (Belgrade)	9.37%	12.5%	−30.91%	−1.85%	[33]
Korea	3.8%	-	−36.48%	-	[37]
Russia	3%	-	−37.28%	-	[3]
Romania	-	1.66%	-	−12.69%	[42]
Northwestern Tunisia	**-**	61.7%	-	+47.35%	[43]
Eastern Spain	-	20.7%	-	+6.35%	[44]

Occurrence of *A. suum* and *M. hirudinaceus* by country, differences compared to Hungarian data.

## Data Availability

Data are contained within the article.
